# Thalidomide for the treatment of angiodysplasia‐related recurrent gastrointestinal hemorrhage: Is low dose a safe and viable option?

**DOI:** 10.1002/ccr3.2501

**Published:** 2019-10-23

**Authors:** Harish Patel, Shehriyar Mehershahi, Danial Haris Shaikh, Jasbir Makker, Sureshkumar Nayudu, Prospere Remy, Sridhar Chilimuri

**Affiliations:** ^1^ Division of Gastroenterology BronxCare Hospital Center a Clinical Affiliate of Mt Sinai Health Systems and Academic affiliate of Icahn School of Medicine Bronx New York; ^2^ Department of Medicine BronxCare Hospital Center a Clinical Affiliate of Mt Sinai Health Systems and Academic affiliate of Icahn School of Medicine Bronx New York

**Keywords:** angiodyslasia and pharmacological management, angiodysplasia and thalidomide, low‐dose thalidomide and GI bleed, thalidomide and small bowel AVM

## Abstract

Thalidomide is often used for the management of refractory gastrointestinal angiodysplasia (GIAD). The tolerance, toxic profile, and compliance of thalidomide are dose‐dependent. The low‐dose thalidomide (50 mg) is safe and a viable option for bleeding related to GIAD.

## Graphical abstract

Thalidomide is often used for the management of refractory gastrointestinal angiodysplasia (GIAD) bleeding. The tolerance, toxic profile, and compliance of thalidomide are dose‐dependent. The low‐dose thalidomide (50 mg) is safe and a viable option for bleeding related to GIAD.

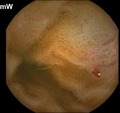

## 1 INTRODUCTION

Gastrointestinal angiodysplasia (GIAD) is the most common cause of obscure gastrointestinal bleeding in the elderly. Endoscopic management of GIAD is often unsuccessful. We present a case of a 75‐year‐old female with GIAD‐related gastrointestinal bleeding and anemia, refractory to endoscopic management. She was managed with low‐dose thalidomide with a favorable response.

Angiodysplasias are the most common vascular malformations of the gastrointestinal tract. These are small, dilated, and tortuous vessels, residing in the mucosa or submucosa.^1^ GIADs are most commonly found in the right colon (78%), followed by the jejunum (10.5%), ileum (8.5%), and duodenum (2.5%).^2^ Though frequently distributed in the colon, GIAD of the small bowel remains the most common source (66%) of obscure gastrointestinal (GI) bleeding,^3^ as diagnosed by video capsule endoscopy (VCE). The etiology of obscure gastrointestinal bleed varies according to age, and GIAD is the most common cause in the elderly (above 50 years of age), while tumors dominate in younger individuals (below 50 years of age).^4^, ^5^

Gastrointestinal angiodysplasia can present as hematochezia, melena, or maroon stools.^6^ They can also present as iron deficiency anemia with intermittently positive occult blood in stools.^7^ In early 1950s, GIAD was managed with hormonal therapy such as estrogens; however, there was no difference in the number of bleeding episodes and transfusion requirements when compared to placebo.^2^ With the innovation of gastrointestinal endoscopy, therapy switched to transendoscopic heat or argon beam coagulation of GIAD. Nevertheless, endoscopic measures remain insufficient in cases of multiple vascular malformations, as lesions are present at numerous sites and the endoscope is limited by its physical maneuvarability.^8^, ^9^ Deep enteroscopy, performed for nonconclusive VCE or for a therapeutic intent after a positive VCE, has a high complication rate (4%).^10^

Recurrent symptoms and requirement for blood transfusions despite endoscopic therapy, necessitate the evaluation for a pharmacological intervention. Medical management in the past comprised of hormonal therapy and octreotide.^11^ The use of the thalidomide for recurrent GIAD is novel. Three prospective trials and one retrospective review provide supporting data.^12^, ^13^, ^14^, ^15^ In view of the limited experience, there is a lack of consensus on the dosage and treatment duration of thalidomide in patients with GIAD. All the trials have used between 100 and 600 mg of thalidomide; however, the medication was withdrawn prematurely due to adverse reactions.^11^ The adverse effects of thalidomide are dose‐dependent.^16^ Lower dose thalidomide may have better tolerance and compliance due to less severe adverse reactions. We present a case of recurrent GIAD, refractory to endoscopic interventions, treated with thalidomide 50 mg for 3 months leading to a 27‐month transfusion free period. The treatment was discontinued prematurely due to intolerance. Clinical experience in United States with thalidomide is scarce. Hence, our report is a novel case registering thalidomide response at a low dose, which can be employed if higher doses are not tolerated.

## 2 CASE PRESENTATION

A 75‐year‐old African‐American woman presented to our emergency department in December 2012 with complaints of generalized weakness and fatigue for 2 weeks. She denied any abdominal pain, nausea, vomiting, or diarrhea. She did not report any rectal bleeding. Her medical comorbidities included hypertension, diabetes mellitus, dyslipidemia and right breast carcinoma for which she underwent a mastectomy a year earlier. Her other surgical history was significant for cholecystectomy in 1990 and a left subclavian to right common carotid bypass for carotid stenosis in 2009. She was a former smoker, consumed alcohol socially, and denied any illicit drug use. She reported allergy to penicillin. Family history was negative for any gastrointestinal malignancy or bleeding. Her medications included aspirin, clopidogrel bisulfate, atorvastatin, metformin, insulin glargine, amlodipine, metoprolol, pantoprazole, ferrous sulfate, and docusate. She was not on any oral anticoagulation or over the counter nonsteroidal antiinflammatory drugs.

In 2011, she had undergone three colonoscopies and two esophagogastroduodenoscopies for evaluation of melanotic stools. The endoscopic studies revealed pan‐diverticulosis and a 5 mm nonbleeding duodenal polyp showing normal intestinal mucosa on histology.

On physical examination, she was pale and lethargic. Bowel sounds were active without any abdominal tenderness or rigidity. A digital rectal exam revealed black guaiac positive stool. Besides lethargy, her review of systems was unremarkable, and vitals revealed a blood pressure of 160/99 mm Hg, heart rate of 90 beats/min, and oxygen saturation of 98% on room air. Laboratory results were significant for a hemoglobin level of 7.3 g/dL with a mean corpuscular volume of 91 fL. Anemia indices reported a serum iron of 31 ug/dL, ferritin of 33.0 ng/mL and a calculated iron saturation of 10.1%. All other laboratory values including platelet count and renal function tests were normal. Echocardiography showed no evidence of aortic valvular disease. Patient received 1 unit of packed red blood cell transfusion.

**Figure 1:**
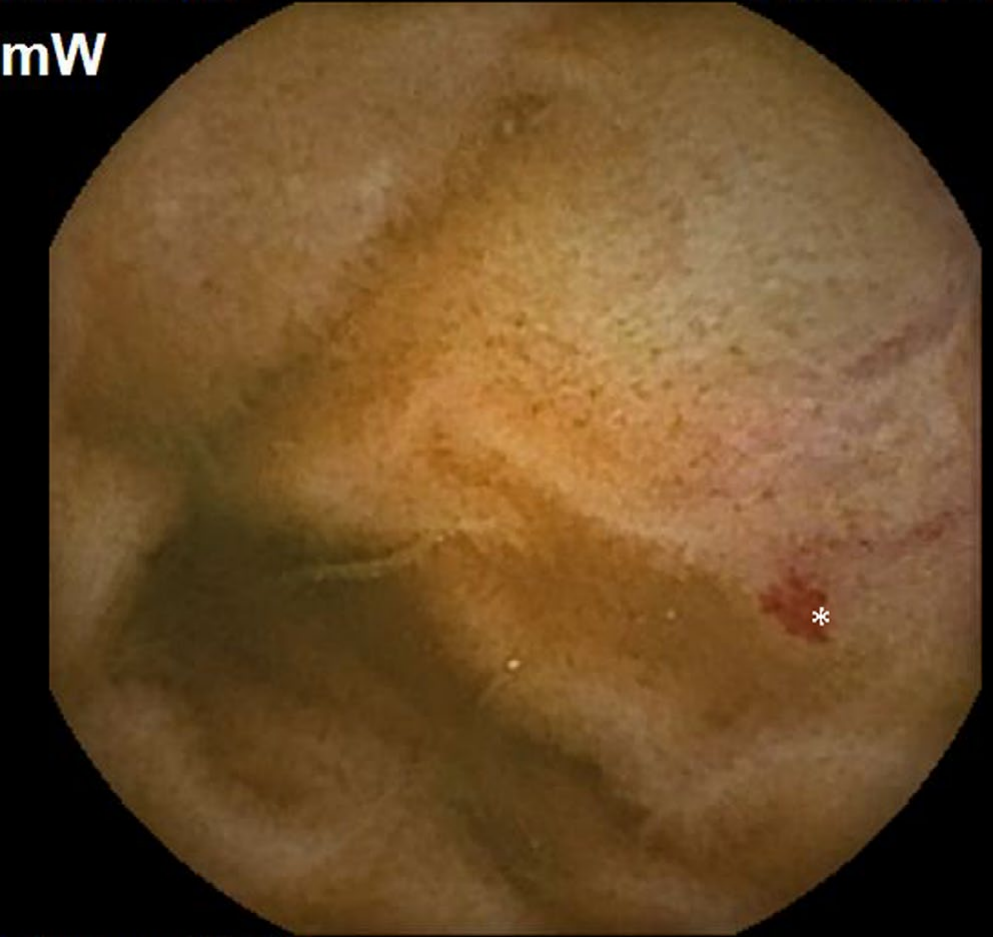
Capsule endoscopy view of small bowel revealing small bowel angiodysplasias

**Figure 2:**
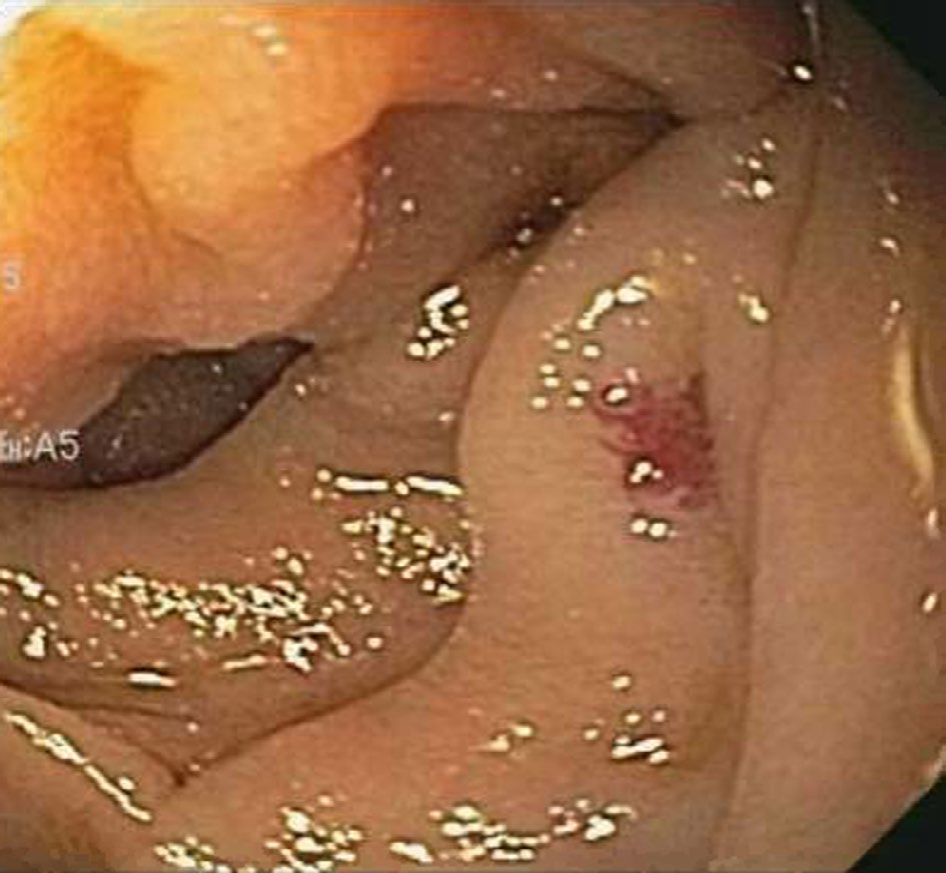
Push enteroscopy view of small bowel revealing angioectatic lesion in the proximal jejunum

**Figure 3:**
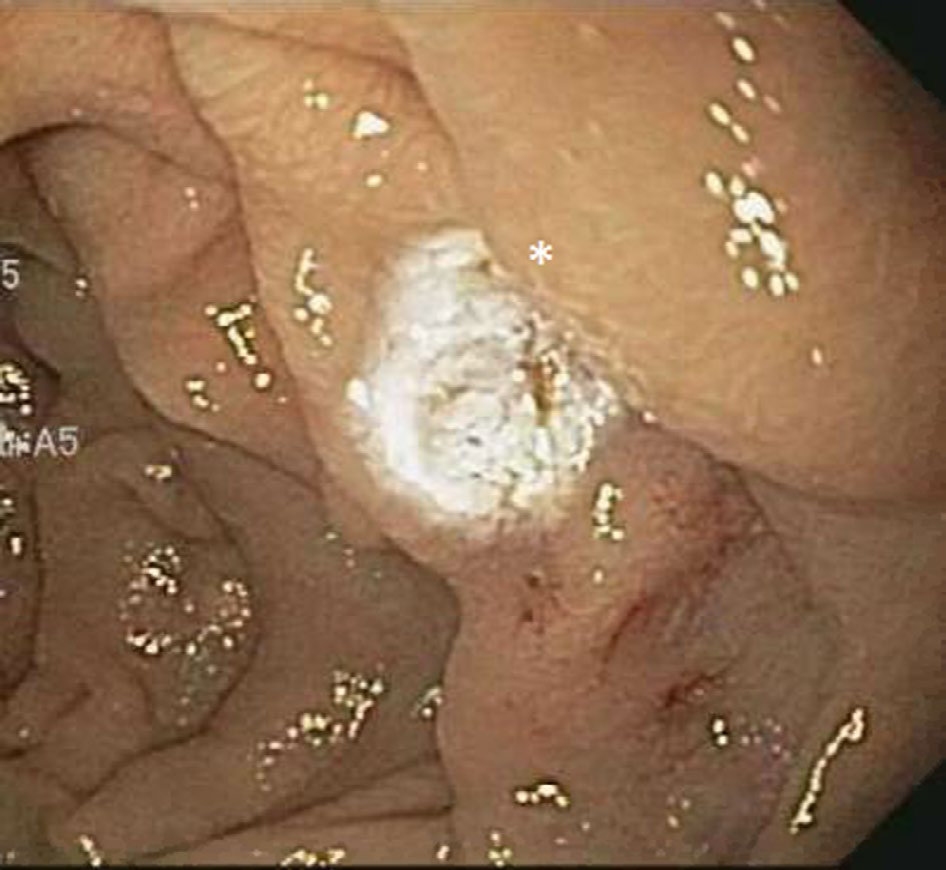
Arteriovenous malformation treated with argon plasma coagulation

Subsequently, she was readmitted a year later with similar complaints and required multiple transfusions. Clopidogrel bisulfate was discontinued due to suspicion of obscure small bowel bleeding. VCE revealed the first duodenal image at 1:04 hour (Figure 1) and small angiodysplasias dispersed in the small bowel from 1:06 to 6:12 hours. The first cecal image was obtained at 7:08 hours. The study deemed small bowel angiodysplasias as a possible etiology for the obscure GI bleed. Gastroscopy with push enteroscopy revealed a bleeding angioectatic lesion in the proximal jejunum, which was treated by argon plasma coagulation (Figure 2 and Figure 3).

Clopidogrel bisulfate cessation did not subside the GI bleed. Over the course of the next 2 years, she had recurrent hospitalizations (8 times) for obscure GI bleed, and required 11 packed red blood cell transfusions, with an average of 3‐4 transfusions every 6 months. In addition, she also underwent a repeat push enteroscopy revealing four small bowel angiodysplasias, treated with argon plasma coagulation assisted thermal therapy.

**Figure 4:**
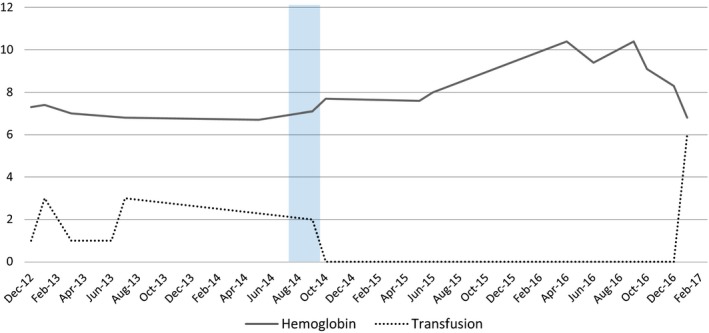
Trend of hemoglobin and transfusion before thalidomide use (December 2012‐ August 2014), treatment duration demonstrated in shaded area (August 2014‐October 2014), and follow‐up (November 2014‐ January 2017)

Pharmacological intervention was considered in 2014 in view of the refractory and recurrent small bowel GIAD. Patient was started on thalidomide 50 mg twice daily after a third push enteroscopy noted two angiodysplasias of the duodenum and jejunum. She remained symptom and transfusion free with stable hemoglobin for the next 27 months (Figure 4), despite self‐discontinuing the drug after 3 months secondary to nausea, vomiting, and constipation. However, major adverse reactions like neuropathy and liver toxicity were not noted.

## 3 DISCUSSION

Thalidomide, a potent antiangiogenic, was first introduced in West Germany in the late 1950s and was marketed for its sedative properties, before the availability of benzodiazepines.^17^ It was prescribed for morning sickness and eventually found to be teratogenic necessitating its withdrawal from the market.^18^ Given safety concerns in pregnancy and side effect of neuropathy, the FDA physician, Frances Kelsey, denied the approval of thalidomide in the United States; hence, it was never marketed.^19^ The pathogenesis of its teratogenicity was not well understood. Rabbit model experiments deemed antiangiogenesis as the etiology of its side effect.^20^ Thalidomide was found to have a potent antiangiogenic effect via downregulation of vascular endothelial growth factor (VEGF).^21^ The drug resurfaced for its antiangiogenic property and was used for the management of erythema nodosum leprosum and vasculitic complications of leprosy.^20^, ^21^ Since then thalidomide, under tighter regulations, has been utilized across multiple specialties, to treat diseases such as HIV‐1‐associated kaposi sarcoma, multiple myeloma, crohn's disease, advanced prostate cancer, and recalcitrant erosive lichen planus.^22^, ^23^, ^24^

Vascular endothelial growth factor expression is increased in patients with colonic angiogenesis, hence playing a significant role in GIAD.^25^ The pathogenesis of increased VEGF in hereditary hemorrhagic telangiectasia^26^ is well known; however, there is a lack of knowledge in other clinical conditions. GIAD has a strong association with many clinical entities. Patients with aortic valve disease (Odds Ratio (OR) 18) and CKD (OR 8.4) have the highest odds of having small intestinal angiodysplasias. Our patient had hypertension, which has an OR of 2.8 for the risk of GIAD.^27^

In a United States‐based pilot study^28^ and a prior randomized control trial on the use of thalidomide^12^ in recurrent GIAD bleeding, patients were selected irrespective of the etiology or the site of the lesion. Ge et al randomly assigned GIAD patients to either 100 mg of thalidomide (n = 28) or 400 mg of iron (n = 27, controls). Rates of response in the thalidomide and control groups were 71.4% and 3.7%, respectively.^12^ Ileum and the jejunum were the most common sites of gastrointestinal bleed. Patients on antithrombotics were not included in the trial. Our patient was on clopidogrel; however, the recurrent bleeding continued despite its cessation; hence, she was considered for thalidomide treatment.

Patients with a decline in VEGF levels with thalidomide treatment had better results in terms of bleeding prevention.^12^ A dose between 100 and 300 mg was considered the optimal dose for antiangiogenesis and demonstrated decrease in VEGF levels.^29^ Suppressing VEGF levels by 25% will have neuropathy related adverse events,^30^ and this can be reversible by decreasing the dose of the medication.^29^ To date, there is no consensus on the optimal dose titration or monitoring of VEGF levels, while managing GIAD with thalidomide.

We performed a Scopus search with TITLE‐ABS‐KEY (angiodysplasia) AND TITLE‐ABS‐KEY (thalidomide) to review the data regarding dosing in various reports. A total of 113 articles populated. A dose of 50 mg thalidomide has been tried in hereditary hemorrhagic telangiectasia‐related gastrointestinal hemorrhage.^31^ Low‐dose thalidomide seems to be a safe, effective, and tolerable option. It has been shown to be valuable in the reduction of epistaxis in patients with hereditary hemorrhagic telangiectasia and in the treatment of chronically‐active, steroid‐dependent crohn's disease.^32^, ^33^ Invernizzi et al demonstrated a decrease in epistaxis in their study population with the use of thalidomide. A response was achieved in 81% of patients at 50 mg/day of thalidomide, 16% of patients at 100 mg/day, and 3% of the patients responded to 150 mg/day. Subjects had only nonserious adverse effects, the most common of which was constipation, followed by drowsiness.^33^ Bauditz et al reported on the utility of thalidomide in patients with steroid‐resistant crohn's disease, where it was observed to have a dramatic improvement in bleeding with a low dose of 100 mg/day.^17^, ^22^

With regards to our patient, we were able to effectively demonstrate a decrease in the need for transfusions and hospitalization with a dose of 50 mg of thalidomide. She responded within 2 weeks of initiating therapy and sustained the effect for over a year. Despite being on a low dose, she suffered rare nonserious side effects, perhaps illustrating less toxic and more tolerable profile of low‐dose thalidomide.

## 4 CONCLUSION

The management of recurrent obscure gastrointestinal bleeding secondary to GIAD is very challenging. The failure of repetitive endoscopic therapy for GIAD necessitates the implementation of medical management. The use of thalidomide for the treatment of GIAD‐related GI bleed is novel but can lead to life‐threatening adverse events. Generally, the response is achieved with a higher dose of thalidomide used for several months. The tolerance and compliance of thalidomide is dose‐dependent due to its toxic profile. Our case sheds light on using a low dose of 50 mg thalidomide for 3 months, which can result in cessation of bleeding and avoid any serious complications associated with treatment.

## INFORMED CONSENT STATEMENT

Patient has provided informed consent for publication of this report and the accompanying images.

## CONFLICT OF INTEREST

The authors declare that they have no conflict of interest.

## AUTHORS' CONTRIBUTION

HKP: has managed patient, reviewed literature, and drafted the manuscript. He has approved the final draft submitted; SM, DS, and JM: has reviewed literature, drafted the manuscript, and approved the final draft submitted; SN and PR: has managed patient, critically reviewed the manuscript, and approved the final draft submitted; SC: helped in planning, contributed to intellectual component  of case report, and reviewed the manuscript. He has approved the final draft submitted.
